# Environmental Control on Microbial Turnover of Leaf Carbon in Streams – Ecological Function of Phototrophic-Heterotrophic Interactions

**DOI:** 10.3389/fmicb.2018.01044

**Published:** 2018-06-04

**Authors:** Jenny Fabian, Sanja Zlatanović, Michael Mutz, Hans-Peter Grossart, Robert van Geldern, Andreas Ulrich, Gerd Gleixner, Katrin Premke

**Affiliations:** ^1^Department Chemical Analytics and Biogeochemistry, Leibniz-Institute of Freshwater Ecology and Inland Fisheries, Berlin, Germany; ^2^Department of Freshwater Conservation, Brandenburg University of Technology Cottbus-Senftenberg, Bad Saarow, Germany; ^3^Department Experimental Limnology, Leibniz-Institute of Freshwater Ecology and Inland Fisheries, Stechlin, Germany; ^4^Institute of Biochemistry and Biology, University of Potsdam, Potsdam, Germany; ^5^GeoZentrum Nordbayern, Department of Geography and Geosciences, Friedrich-Alexander University Erlangen-Nuremberg (FAU), Erlangen, Germany; ^6^Institute of Landscape Biogeochemistry, Leibniz Centre for Agricultural Landscape Research, Müncheberg, Germany; ^7^Department of Biogeochemical Processes, Max Planck Institute for Biogeochemistry, Jena, Germany

**Keywords:** algae, bacteria, microbial interactions, ^13^C stable isotopes, PLFA, terrestrial carbon, streambed structure, light

## Abstract

In aquatic ecosystems, light availability can significantly influence microbial turnover of terrestrial organic matter through associated metabolic interactions between phototrophic and heterotrophic communities. However, particularly in streams, microbial functions vary significantly with the structure of the streambed, that is the distribution and spatial arrangement of sediment grains in the streambed. It is therefore essential to elucidate how environmental factors synergistically define the microbial turnover of terrestrial organic matter in order to better understand the ecological role of photo-heterotrophic interactions in stream ecosystem processes. In outdoor experimental streams, we examined how the structure of streambeds modifies the influence of light availability on microbial turnover of leaf carbon (C). Furthermore, we investigated whether the studied relationships of microbial leaf C turnover to environmental conditions are affected by flow intermittency commonly occurring in streams. We applied leaves enriched with a ^13^C-stable isotope tracer and combined quantitative and isotope analyses. We thereby elucidated whether treatment induced changes in C turnover were associated with altered use of leaf C within the microbial food web. Moreover, isotope analyses were combined with measurements of microbial community composition to determine whether changes in community function were associated with a change in community composition. In this study, we present evidence, that environmental factors interactively determine how phototrophs and heterotrophs contribute to leaf C turnover. Light availability promoted the utilization of leaf C within the microbial food web, which was likely associated with a promoted availability of highly bioavailable metabolites of phototrophic origin. However, our results additionally confirm that the structure of the streambed modifies light-related changes in microbial C turnover. From our observations, we conclude that the streambed structure influences the strength of photo-heterotrophic interactions by defining the spatial availability of algal metabolites in the streambed and the composition of microbial communities. Collectively, our multifactorial approach provides valuable insights into environmental controls on the functioning of stream ecosystems.

## Introduction

The decomposition of organic matter (OM) by microbial sediment biofilms contributes substantially to energy flow and nutrient cycling in fluvial ecosystems (Cole and Caraco, 2001; Hotchkiss et al., 2014). Besides *in situ* OM production, terrestrial OM often dominates OM input in streams, with falling leaves being the major form of external particulate OM input (Fisher and Likens, 1973). Owning to its complex, aromatic molecular structure and distinct chemical composition, the bioavailability of terrestrial OM to microbial communities is considered to be limited (Farjalla et al., 2009; Guillemette et al., 2013). Yet, multiple factors, including climate, streambed hydrology, primary production, nutrient availability, and microbial biomass and composition, modulate the relevance of molecular properties for the availability of organic compounds (e.g., Tank et al., 2010; Ziegler and Lyon, 2010; Singer et al., 2011; Rier et al., 2014). Therefore, it is widely accepted that microbial decomposition of OM is related to environmental conditions rather than the molecular structure of the substrate (Schmidt et al., 2011; Marín-Spiotta et al., 2014).

Biofilms dominate microbial life in streams and form highly diverse and complex matrix-enclosed communities in and upon the streambed as well as on OM fragments (Battin et al., 2007). In subsurface streambeds, i.e., the hyporheic zone, biofilms are dominated by heterotrophic species. In light mediated streambed areas, i.e., the benthic zone, eukaryotic algae and cyanobacteria, together with heterotrophic bacteria and fungi form diverse biofilms that favor potential metabolic interactions (Rier et al., 2007; Danger et al., 2013). Algal exudation of dissolved OM provides a highly bioavailable substrate to microbial heterotrophs that is selectively removed from the OM pool (Guillemette et al., 2013). These organic compounds of phototrophic origin are rich in energy as well as nutrients and therefore are assumed to stimulate the metabolic capability of fungi and heterotrophic bacteria to decompose less bioavailable terrestrial OM (Kuehn et al., 2014). Accordingly, interactions among microbial photo- and heterotrophs significantly alter the turnover of terrestrial OM in aquatic ecosystems (Rier et al., 2007; Danger et al., 2013). Yet, additional research implies that fungi and bacteria do not equally respond to the availability of algal OM, with stimulating effects on activity and biomass mainly observed for bacteria (Mille-Lindblom and Tranvik, 2003; Frossard et al., 2012; Fabian et al., 2017). Nevertheless, although environmental conditions apparently influence the appearance and strength of photo-heterotrophic interactions (Rier et al., 2014; Hotchkiss and Hall, 2015), it remains unclear how multiple environmental factors synergistically affect their significance for microbial turnover of terrestrial OM in streams.

In stream ecosystems, microbial turnover of OM is significantly influenced by hyporheic flow, that is the flow of surface water through the pore space of the streambed in flow paths that return to surface water (Battin et al., 2003; Boano et al., 2014; Perujo et al., 2017). The hyporheic flow varies according to the distribution and spatial arrangement of sediment grain sizes (Bear, 1972), or in other words the structure of the streambed. In natural ecosystems, the arrangement of the different sediment grain sizes varies both vertically and horizontally and poorly conductive habitats of sand or silt commonly alternate with well-conducting habitats of gravel or cobbles (Powell et al., 2005; Bridge, 2009). These spatial variations in hydraulic conductivity cause up-and-down welling of water across the streambed surface, thus increasing the hyporheic flow, which in turn promotes the transport of dissolved compounds through the streambed. (Malard et al., 2002; Salehin et al., 2004).

Accordingly, streambed structure influences the hyporheic transport of dissolved organic resources from the benthic zone into the pore space of deeper sediment layers (Battin, 2000; Navel et al., 2011). Previous research underlines that changes in the spatial distribution of resources cause variations in resource use efficiency and organic carbon (C) turnover in streambed biofilms (Battin et al., 2003; Singer et al., 2010, 2011). Hence, streambed structure likely determines the extent to which streambed heterotrophs interact with OM of phototrophic origin during C turnover through associated changes in the hyporheic flow of phototrophic compounds through the streambed.

Given the multitude of environmental factors whose influence on microbial C turnover has been reported for streambed biofilms, the ecological relevance of photo-heterotrophic interactions for the degradation of terrestrial OM may vary greatly among different ecosystem types (Wagner et al., 2017). In intermittent streams, flow disruption provides a strong influence on microbial function in ecosystem processes (Febria et al., 2015; Duarte et al., 2017) and the turnover of terrestrial OM is largely determined by the characteristic dry-wet cycles (Abril et al., 2016; Weise et al., 2016). It is therefore likely that light conditions, as well as associated photo-heterotrophic interactions, are less important for the turnover of terrestrial OM in intermittent streams than assumed for perennial streams. However, given the significant contribution of intermittent streams to global C fluxes (Tockner and Stanford, 2002), it is important to understand how recognized functions of photo-heterotrophic interactions for terrestrial OM turnover in perennial streams can be transferred to these temporary waters.

This study investigated how light and streambed structure interactively alter the cycling of terrestrial OM through the microbial food web in temporary stream ecosystems with a focus on possible metabolic interactions between microalgae and heterotrophic bacteria. In experimental streams, we manipulated hyporheic flow and phototrophic activity by applying different levels of streambed structure and light intensities, respectively. Fragments of leaf litter enriched in ^13^C stable isotopes were applied as a proxy for terrestrial OM, whose cycling through the microbial food web was followed via isotope analysis of different C pools: microbial biomass [phospholipid derived fatty acids (PLFA), dissolved organic carbon (DOC), and respired C]. The findings were related to treatment-related variations in overall biofilm community composition derived from PLFA, as well as bacterial biofilms, as analyzed by terminal restriction fragment length polymorphism (T-RFLP) profiling. Based on findings in perennial ecosystems, we expected that bacterial assimilation of leaf C is stimulated by phototrophic activities, and therefore, related to light conditions. In addition, we assume that streambed structure defines the hyporheic transport of phototrophic metabolites through the streambed. Accordingly, we hypothesize that effects of light on leaf C turnover correlate with streambed structure.

## Materials and Methods

### Study Design

**FIGURE 1:**
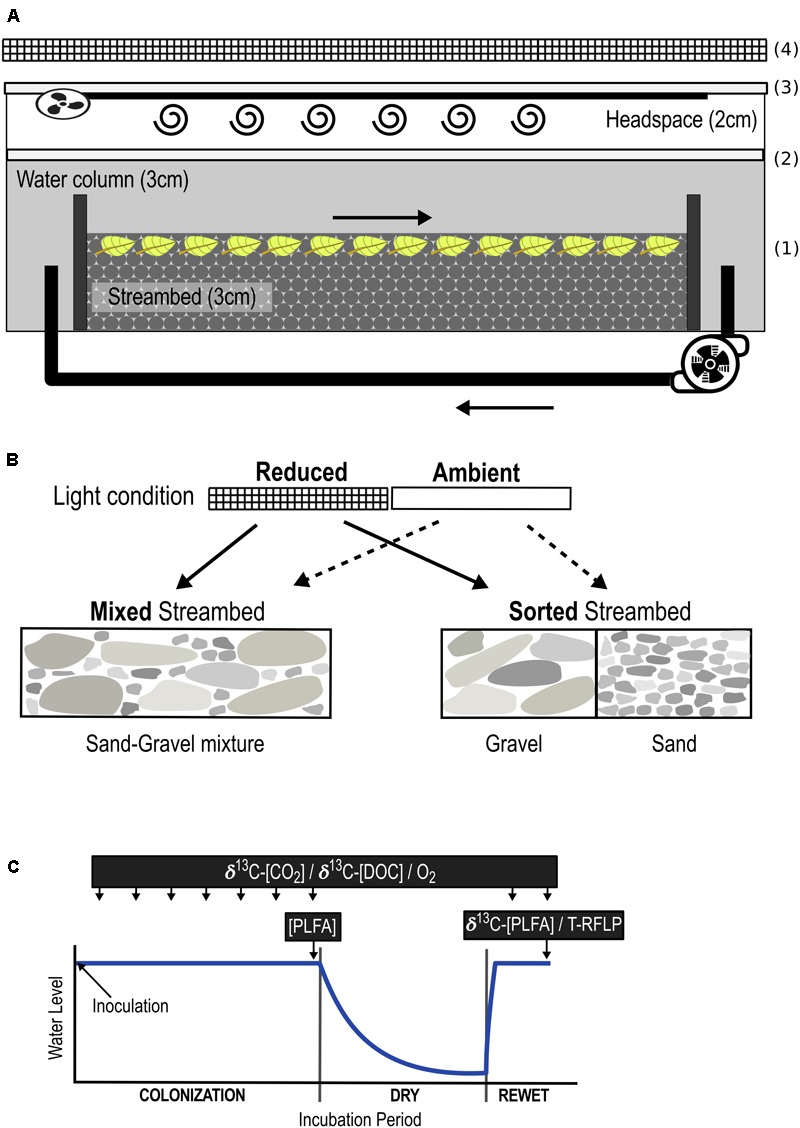
Overview of the experimental set-up. Experimental outdoor streams **(A)** were filled with a streambed layer of gravel and sandy sediments in two different arrangements: sorted and mixed **(B)**. ^13^C-enriched leaf fragments were mixed into the upper 1 cm of the streambed (1). Streams were sealed gas-tight without headspace for oxygen measurements (2), and with headspace for the analysis of headspace CO_2_ (3). A fan attached to the lid and connected to a 3-m tube facilitated mixing of the headspace air. Streams were exposed to ambient (outdoor condition) and reduced (54 ± 8% of ambient) light intensities, the latter achieved by placing 3 layers of black mosquito net 20 cm above the streams (4). The combinations of both light availability and streambed structure added up to 4 different treatments: (1) Sorted Streambed + Reduced Light, (2) Sorted Streambed + Ambient Light, (3) Mixed Streambed + Reduced Light, (4) Mixed Streambed + Ambient Light. **(C)** Streams were inoculated with a natural microbial community and then incubated for a 7-week colonization period, followed by a 6-week dry and a 2-week rewet period. Quantity of respired C and DOC were measured over the entire incubation period. The composition of the biofilm community (PLFA/T-RFLP) was measured at the end of the colonization and rewet period. The stable isotopic composition of biofilm fatty acids was measured at the end of the rewet period.

Applying a 2 × 2 factorial design, we manipulated streambed sediment composition and light in 16 outdoor, experimental streams, i.e., flume mesocosms (400 × 12 × 10 cm, **Figure 1A**), installed beneath a white tent to prevent uncontrolled input of coarse OM and rainwater. Streams were filled with a 3-cm streambed of sand and gravel, which were rinsed with 10% hydrochloric acid and tap water prior to use to avoid uncontrolled microorganism input. As a proxy for terrestrial OM, 31.25 g C m^-2^ of ^13^C-labeled beech leaf fragments were mixed into the upper 1 cm of the streambed. A surface-water column of 2.9 ± 1 cm [tap water + nitrogen (4,54 mg L^-1^ N-NO_3_^-^) and phosphorus (542 μg L^-1^ P-PO_4_^-3^) + 0.3 ml/L SL-10 trace element solution, according to Lehman (1980)] was circulated at a constant flow velocity of 2.5 ± 0.03 cm s^-1^ using an aquarium pump (EHEIM GmbH & Co. KG, Deizisau, Germany). Evaporated water was replaced with deionized water.

Streambed sediments were inoculated with a microbial community (16.45 mg CL^-1^) sampled from a first-order rarely intermittent stream (Waldbach, Bad Saarow, Germany, N52° 16.4578 E14° 3.2794) located in a forest catchment. The inoculum was generated from a suspension in stream water of randomly collected surface sediments, leaf litter, and woody fragments for 48 h followed by filtration through a 125-μm mesh to exclude macro-invertebrates and larger OM particles. After 7 weeks of colonization, we ran a 6-week dry/2-week rewet cycle to generate conditions of an intermittent stream ecosystem (Larned et al., 2010). Primary production was manipulated by two levels of light intensity: ambient (outdoor conditions) vs. reduced light. For the latter condition, ambient light intensity was reduced by 54 ± 8% by placing 3 layers of black mosquito net (grid size 1.29 × 1.13 mm) 20 cm above the stream. Differences in streambed structure and associated changes in hyporheic flow were generated by varying the structural arrangement of gravel (2–10-mm grain size, d50 = 4.75 mm) and sand (0.2–2-mm grain size, d50 = 0.57 mm) in the streambed. Solute transfer in gravel greatly exceeds that in sand and sand-gravel (Mendoza-Lera et al., 2017). As a result, alternating patches of sand and gravel intensify up- and down-welling, leading to enhanced vertical water exchange (VWE) and thus, solute transport into the pore spaces of sorted as compared to homogenously mixed streambeds (Malard et al., 2002). Accordingly, we generated two levels of streambed structure by filling 8 streams with a homogeneous mixture of sand and gravel (Mixed Streambed, reduced solute transport) and 8 streams with 8 alternating blocks (45 cm) of gravel and sand (Sorted Streambed, enhanced overall solute transport).

The combination of both factors, light intensity and streambed structure, added up to 4 different treatments, with 4 replicates each (**Figure 1B**). Treatment-related effects on microbial C respiration and DOC production, relate to whole stream scale and were measured weekly throughout the entire experimental phase (**Figure 1C**). Treatment-related effects on the function and composition of biofilm community relate to sediment scale, thus gravel, sand, and sand-gravel sediments were sampled and analyzed separately. Biofilm community composition was measured at the end of the colonization and rewet period. The stable isotope composition of biofilm fatty acids was measured at the end of the rewet period.

### Monitoring of Treatment Conditions

Light intensity at the streambed and water temperature were monitored every 10 min (ONSET HOBO Pendant^®^ Temperature/Light 64K Data Logger, PCB Synotech GmbH, Hückelhoven, Germany). To monitor solute transfer through the streambed, surface water flow and VWE were estimated by using the conservative fluorescent tracer uranine, following Mutz et al. (2007). A small pulse of concentrated tracer solution (3 μg L^-1^) was added to the stream at the discharge side and the tracer concentration in the water column was measured every 15 min for 6 h at the opposite end of the stream with a fluorometer (LLF-M, Fiber-Optic fluorometer; Gotschy Optotechnik, Adnet, Austria). Surface water flow was calculated based on the tracer’s mean travel time. VWE was determined from the decrease in uranine concentration resulting from down-welling of the tracer into the streambed and upwelling into the free water. Measurements were made at night and under stable pH to avoid photochemical decay and quenching of the tracer.

### Quantification and Stable Isotope Analysis of DOC and Respired C

For DOC quantification and isotope analysis, filtered surface water samples (cellulose acetate, 0.45 μm; Sartorius Stedim Biotech GmbH, Göttingen, Germany) were fixed with 22 mmol L^-1^ ZnCl_2_ and stored at 4°C until analyzed on an OI Analytical Aurora 1030W TIC-TOC analyzer (OI Analytical, College Station, TX, United States) coupled in continuous flow mode to a Thermo Scientific Delta V *plus* IRMS. Analysis of internal laboratory standards enabled an analytical precision of <±0.3‰ for δ^13^C-DOC (±1σ). Isotope ratios are expressed as *δ*^13^C in per mil (‰) (Coplen, 2011) relative to the Vienna PeeDee Belemnite (VPDB) standard.

Microbial C respiration was calculated from day–night dynamics of O_2_ in the water column. Streams were sealed headspace-free with acrylic glass lids, and O_2_ was measured every 30 min for 24 h using a multichannel fiber oxygen optode system (Oxy 10 mini; Presens, Regensburg, Germany). C respiration rates were calculated and standardized to 20°C according to Mendoza-Lera and Mutz (2013) and expressed as C equivalents assuming a respiratory quotient of 0.85 (Bott, 2007). The isotopic composition of respired C was obtained by Keeling plot analysis of CO_2_ emitted from the water column into the headspace during night incubations (10 PM to 4 AM) following Pataki et al. (2003). To this end, streams were sealed gas-tight with acrylic glass lids and equipped with an automatic sampling system for headspace CO_2_ connected to a CO_2_-isotope analyzer (Off-Axis ICOS CCIA; Los Gatos Research, San Jose, CA, United States) that facilitated online monitoring of CO_2_ concentration and δ^13^C-CO_2_ values every 2 h throughout incubation, modified after Fabian et al. (2017). A fan (ACT-RX Technology Corporation, Taiwan; airflow capacity of 116 L min^-1^) attached to the insides of the lids and connected to a 3-m tube enabled homogenous mixing of the headspace during incubations. Regular measurements of an internal laboratory CO_2_ gas standard (0.15% CO_2_ in 70% N_2_ + 30% O_2_) allowed for data correction with respect to instrumental drift and linearity during measurements, yielding a precision of 2‰ for *δ*^13^C-CO_2_.

### Biofilm Community Composition Analysis

PLFA are present in the membranes of all living cells, but rapidly degrade to neutral lipids upon cell death (White et al., 1979; Willers et al., 2015). Hence, monitoring the variation in PLFA allows sensitive and reproducible characterization and quantification of microbial communities (Boschker and Middelburg, 2002; Yao et al., 2015). PLFA were extracted from 0.2 g of freeze-dried leaf fragments sampled at the end of the experiment, followed by solid-phase separation from other lipids (Bond Elute LRC cartridge 500 mg; Agilent Technologies, Santa Clara, CA, United States) and mild alkaline methylation to fatty acid methyl esters (FAMEs) (White and Tucker, 1969; Frostegård et al., 2011; Steger et al., 2011). FAMEs were quantified on a GC-MS system (Agilent 6890/Agilent 5973, Agilent Technologies, Germany) using a CP SIL 88 column (100 m, ID: 0.25 mm, film: 0.2 μm; Agilent Technologies), as described in Boëchat et al. (2014). A standard mix (Supelco 37 Component FAMEs Mix) was applied for system calibration. FAMEs were identified by their mass spectra and retention times, which were validated with standard FAMEs or by comparing their equivalent chain lengths with literature reports for the applied column (Eder, 1995; Santercole et al., 2012).

For more detailed profiling of bacterial biofilm communities, DNA was extracted from freeze-dried leaf fragments sampled at the end of the experiment using the NucleoSpin Soil kit (Macherey-Nagel, Germany). T-RFLP profiling was performed as described by Weise et al. (2016). GeneMapper software version 5.0 (Applied Biosystems) was used for data analysis. T-RFLP profiles were normalized according to Dunbar et al. (2001).

### Stable Isotope Analysis of Biofilm Lipids

FAME isotope ratios, expressed as δ^13^C in ‰ relative to VPDB, were measured on a HP5890 GC (Agilent Technologies, Palo Alto, CA, United States) connected to a Delta plus XL IRMS via a combustion interface GC/C III (both Thermo Scientific, Bremen, Germany) following Kramer and Gleixner (2008). Based on previous culture observations (de Carvalho and Fernandes, 2010; Akinwole et al., 2014; Arce Funck et al., 2015; Strandberg et al., 2015), cell membrane fatty acids, of which we could analyze the carbon isotope ratios, were grouped into bacterial (*iso*14:0, *iso*15:0, *anteiso*15:0, *iso*16:0, *iso*17:0, *cyclic*17:0), algal (C20:4w6, C20:5w3, C22:5w6, C22:6n3), or fungal + algal (C17:1w9, C18:1w7, C18:2w6, C18:3w3, C18:3w6, C18:1w9) PLFA biomarker. Further, we identified several unspecific, universal PLFA (C14:0, C15:0, C16:0, C16:1w5, C16:1w7, C17:0, C18:0). Consequently, to link identity and function of microbial decomposers, we focused on the biomarkers specific for heterotrophic bacteria, as biomarkers for fungi overlap with those for algae.

### Calculation of the Proportion of Leaf C in Organic and Respired C Pools

The fraction of leaf C in organic and respired C pools (*F_Leaf_*) was calculated by applying a two end-member mixing model as follows (1), assuming two C substrate pools in the system:

(1) $$F_{Leaf}\text{​}\left[\%\right]=\frac{\left(\delta^{13}{\text{C}}_{Sample}-\delta^{13}{\text{C}}_{Atmospheric}\right)}{\left(\delta^{13}{\text{C}}_{Leaf}-\delta^{13}{\text{C}}_{Atmospheric}\right)}\times 100$$

The first C pool (*δ^13^C_Atmospheric_*) comprised all C substrates that originally entered the ecosystem C cycle through photosynthetic fixation of atmospheric CO_2_, representing natural isotope composition. *δ^13^C_Atmospheric_* was therefore assumed to equal 1‰ and -23‰ in calculations for the respired and organic C pools, respectively (Peterson and Fry, 1987; Finlay, 2004). The second C substrate pool (*δ^13^C_Leaf_*) comprised C that originally entered the ecosystem C cycle through the decomposition of leaf OM enriched in ^13^C isotope. *δ^13^C_Leaf_* was obtained from isotope analysis of 6 subsamples taken from the initial pool of leaf fragments and equaled 804 ± 12‰.

For DOC pools, *δ^13^C_Sample_* was obtained by isotope analysis assuming isotope fractionation during microbial C degradation to be negligible (Mary et al., 1992). For biofilm C respiration, *δ^13^C_Sample_* was obtained by Keeling plot analysis (Keeling, 1961; Pataki et al., 2003) of respired CO_2_, corrected according to Mook et al. (1974). For biofilm C assimilation, *δ^13^C_Sample_* was obtained by isotope analysis of PLFA corrected according to Boschker and Middelburg (2002). The quantity of leaf derived C ([*C_Leaf_*]) for each C pool was obtained by multiplying *F_Leaf_* with the total quantity of the respective C pool ([*C_Sample_*]), according to

(2) $$[{\text{C}}_{Leaf}]\;=\;[{\text{C}}_{Sample}]\;\times \;F_{Leaf}.$$

### Statistical Analysis

Analyses were performed with the statistical software (R Core Team, 2016) using the packages nlme, vegan, MASS, multcomp, and psych, at a significance level of *P* ≤ 0.05. Significance of environmental factors for the composition of organic C pools as well as biofilm composition and C metabolism was evaluated by computing a linear mixed-effects model for each studied parameter. We included the treatments “Light” (2 levels: Reduced versus Ambient) and either “Streambed Structure” (2 levels: Sorted versus Mixed) or “Sediment” (3 levels: Gravel, Sand, Sand-Gravel) as fixed effects. For time series (DOC/C respiration), we included “Sampling Day” as a random effect to correct for pseudo-replication, i.e., re-sampling of the same stream at different dates. To test for an interaction effect between the drought event and the treatments on the respective parameter, we included “Period” as a fixed effect. In the respective model, we further included “Stream” as a random effect to allow a different intercept for each replicate. Interactions among fixed effects were tested for each model and were included if significant. The statistical significance of each fixed effect was tested using a likelihood-ratio (LR) test by comparing the model with and without the target effect.

Relationships between the composition of the microbial (PLFA) and bacterial (T-RFLP of the 16S rRNA gene) communities and the environmental variables (light availability, habitat) and respired C were investigated by distance-based redundancy analysis (dbRDA). Accordingly, a distance matric was generated by calculating pairwise Bray–Curtis dissimilarities between individual replicate samples from square root-transformed PLFA or T-RFLP data, followed by principle coordinate analysis (PCoA). The eigenvalues obtained in PCoA were then visualized in a redundancy analysis (RDA). In the following, the significance of the contribution of a particular variable to the explained variation was tested by permutational multivariate analysis of variance (PERMANOVA), applying 500 permutations and a significance level of *P* > 0.05. The resulting pseudo-*F*-values and significance level were reported for each test result.

## Results

### Treatment Conditions

Mean light availability was significantly higher under ambient, (44.2 ± 4.4) μmol m^-2^ h^-1^, than reduced, (26.2 ± 1.0) μmol m^-2^ h^-1^, light conditions. Pore water exchange in gravel blocks occurred within 10 min after tracer addition, which was below the 15-min measuring interval. Therefore, VWE in sorted streambeds was calculated based on the pore water exchange in the sandy sediment habitats. The VWE was significantly higher in the sandy sediments of sorted streambeds, (3.80 ± 0.65) dm^3^ m^-2^ h^-1^, than in the sand-gravel sediments of homogenously mixed streambeds, (3.08 ± 0.16) dm^3^ m^-2^ h^-1^, (*LR* = 6.50, *P* = 0.01). Variations in VWE were independent of light conditions (*LR* = 0.01, *P* = 0.91).

### Biofilm Cycling of Leaf C Within Respired C and DOC Pools

Quantitative changes in microbial C turnover were derived from variations in DOC concentrations and C respiration rates. In addition, variations of the leaf C fraction in the DOC and respired C pools, *F_Leaf_*, provide information about the proportional use of leaf C compared to other C sources. Accordingly, the combination of quantitative analyses with stable isotope analyses provides information on whether a change in C turnover is associated with altered use of leaf C within the microbial food web.

**FIGURE 2:**
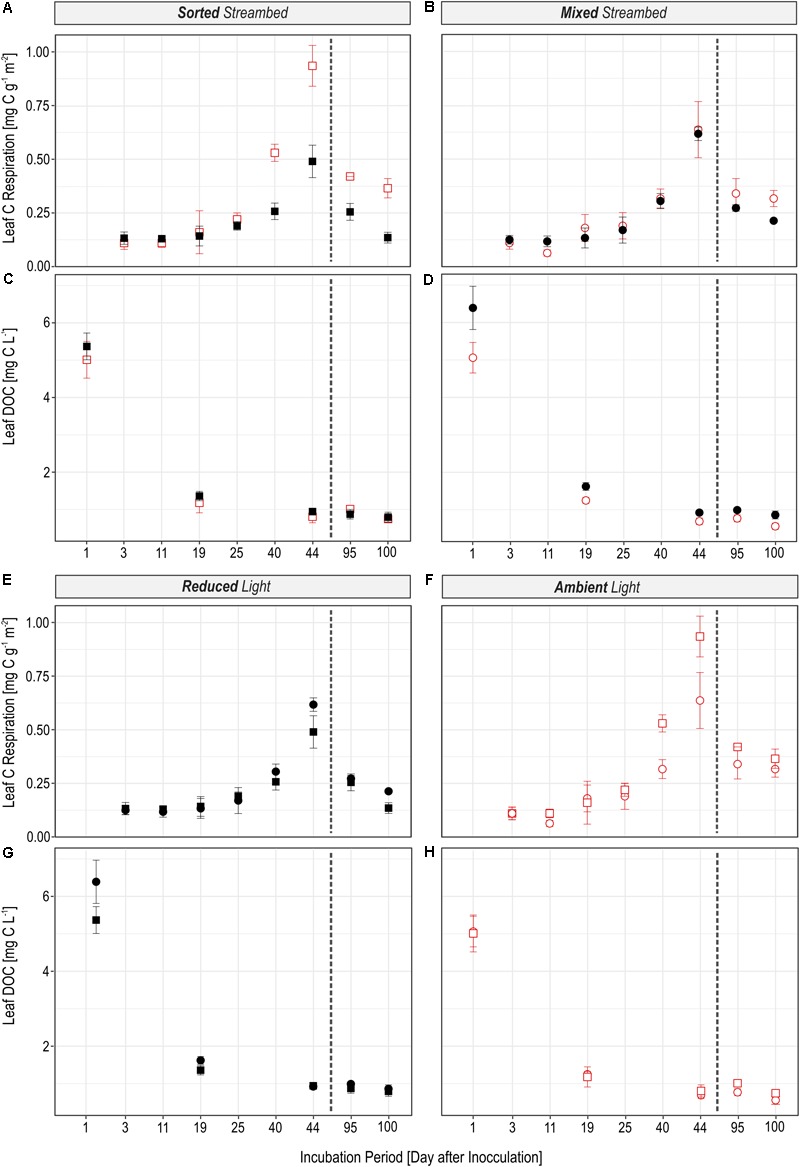
Figures illustrate the effect of the light availability **(A–D)** and the effect of the streambed structure **(E–H)** on the magnitude of microbial leaf C turnover. The amount of leaf C which was respired per hour **(A,B,E,F)** and which was bound in the DOC pool **(C,D,G,H)** was different in experimental streams depending on whether the streambed structure was sorted (squares) or homogeneously mixed (circles) and whether streams were incubated under reduced (black) or ambient light conditions (red). The dashed line separates pre- and post-drought phases.

**FIGURE 3:**
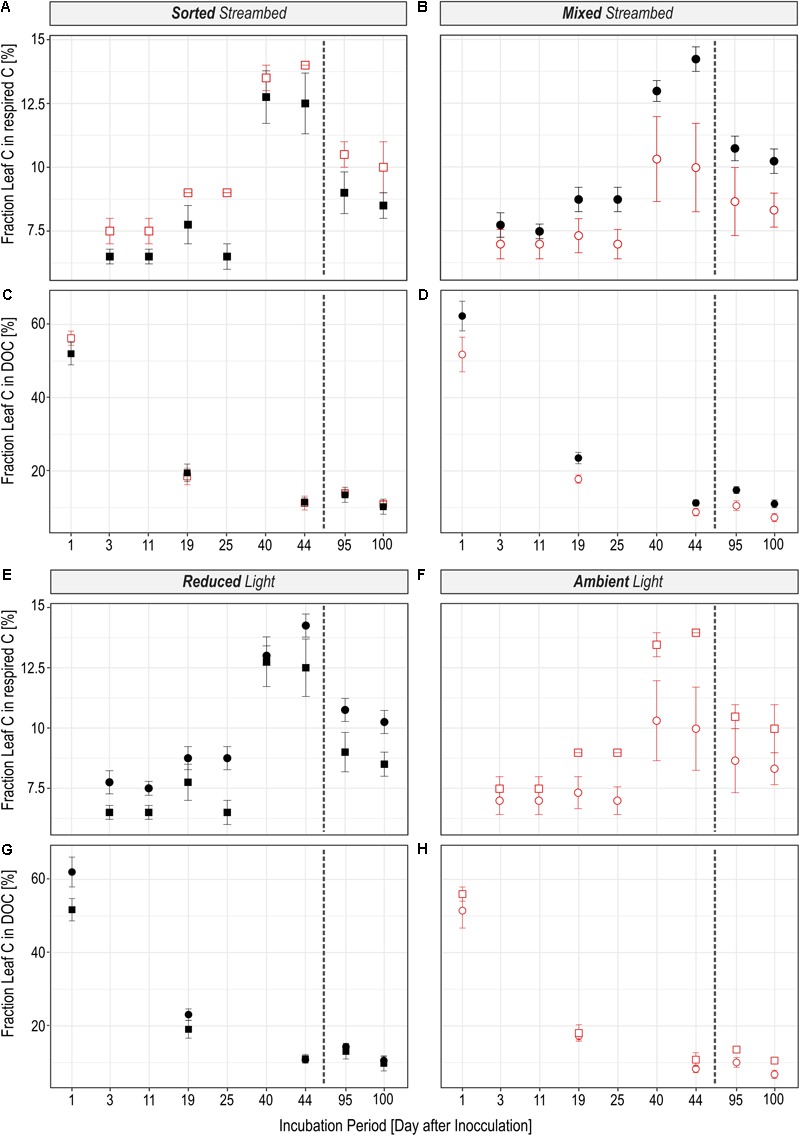
An overview is given about the fraction by which leaf C is utilized in streams with a sorted (squares) or homogenously mixed (circles) streambed structure that were either incubated under reduced (black) or ambient (red) light conditions. **(A–D)** Illustrate the effect of the light availability and **(E–H)** the effect of the streambed structure on the fractional use of leaf C in respired C **(A,B,E,F)** and in DOC **(C,D,G,H)**. The dashed line separates pre- and post-drought phases.

The rate to which leaf C was respired was significantly regulated by light availability (**Figures 2A,B**; *LR* = 16.72, *P* < 0.0001), independent of streambed structure (**Figures 2E,F**; *LR* = 0.02, *P* = 0.88). On the contrary, we observed a significant interaction between light availability and streambed structure on *F_Leaf_* in respired C (**Figures 3A,B**; *LR* = 18.89, *P* < 0.0001). Hence, the proportion of leaf C in respired C was higher under ambient than under reduced light conditions in sorted streambeds, whereby opposing effects were observed in mixed streambeds. Furthermore, under ambient light, the proportion of leaf C in respired C was significantly higher in sorted than in mixed streambeds (**Figures 3E,F**; *P* < 0.001).

Similar to the respired C pool, we observed a significant interaction between streambed structure and light availability for treatment-induced changes in *F_Leaf_* in the DOC pool (**Figures 3C,D**; *LR* = 11.57, *P* < 0.001). Accordingly, the proportion of leaf C in the DOC pool was significantly lower under ambient than under reduced light conditions in homogenously mixed streambeds (**Figures 2C,D**; *P* = 0.001). On the contrary, the proportion of leaf C did vary according to light availability among DOC pools of sorted streambeds (*P* = 0.5). Under ambient light conditions, the proportion of leaf C in DOC was significantly higher in sorted than in mixed streambeds (**Figures 3G,H**; *LR* = 5.21, *P* = 0.02).

Observed treatment effects on the rate of leaf C respiration and on the quantity of leaf DOC did not differ before and after the drought event *(Light × Phase: LR* = 0.15, *P* = 0.70, *Streambed × Phase: LR* = 0.03, *P* = 0.86). The same accounts for the effect of our treatments on the proportion of leaf C in the respired C and the DOC pool.

### Biofilm Assimilation of Leaf C

**FIGURE 4:**
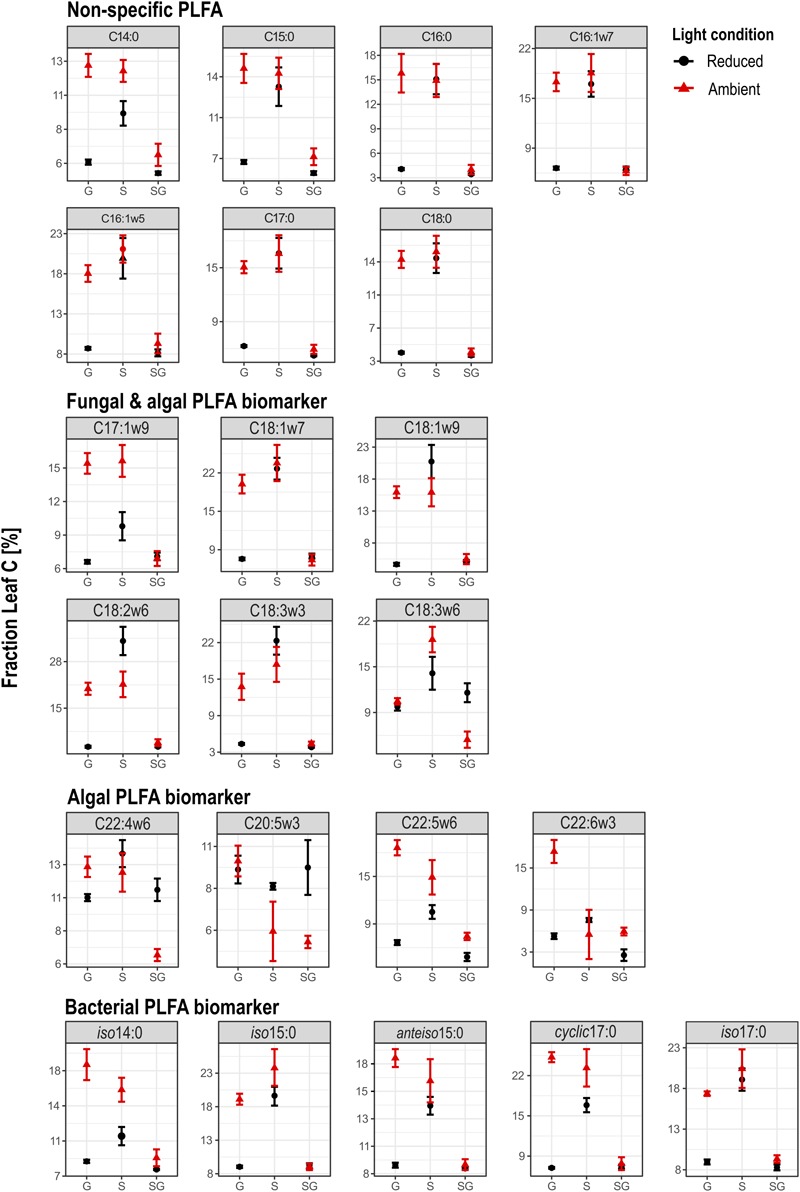
Overview on the fraction of leaf C in PLFA biomarkers of heterotrophic bacteria, microalgae and fungi. G, Gravel; S, Sand; SG, Sand-Gravel.

Carbon isotopes could be analyzed from 22 identified PLFA biomarkers. Within the group of non-specific PLFAs and bacteria-specific PLFAs, variations in *F_Leaf_*, hence the proportion of leaf C in PLFA biomass, were similar in terms of light availability and streambed structure (**Figure 4**). In contrast, *F_Leaf_* values of algae-specific PLFA biomarkers varied differently relative to the treatment conditions.

**FIGURE 5:**
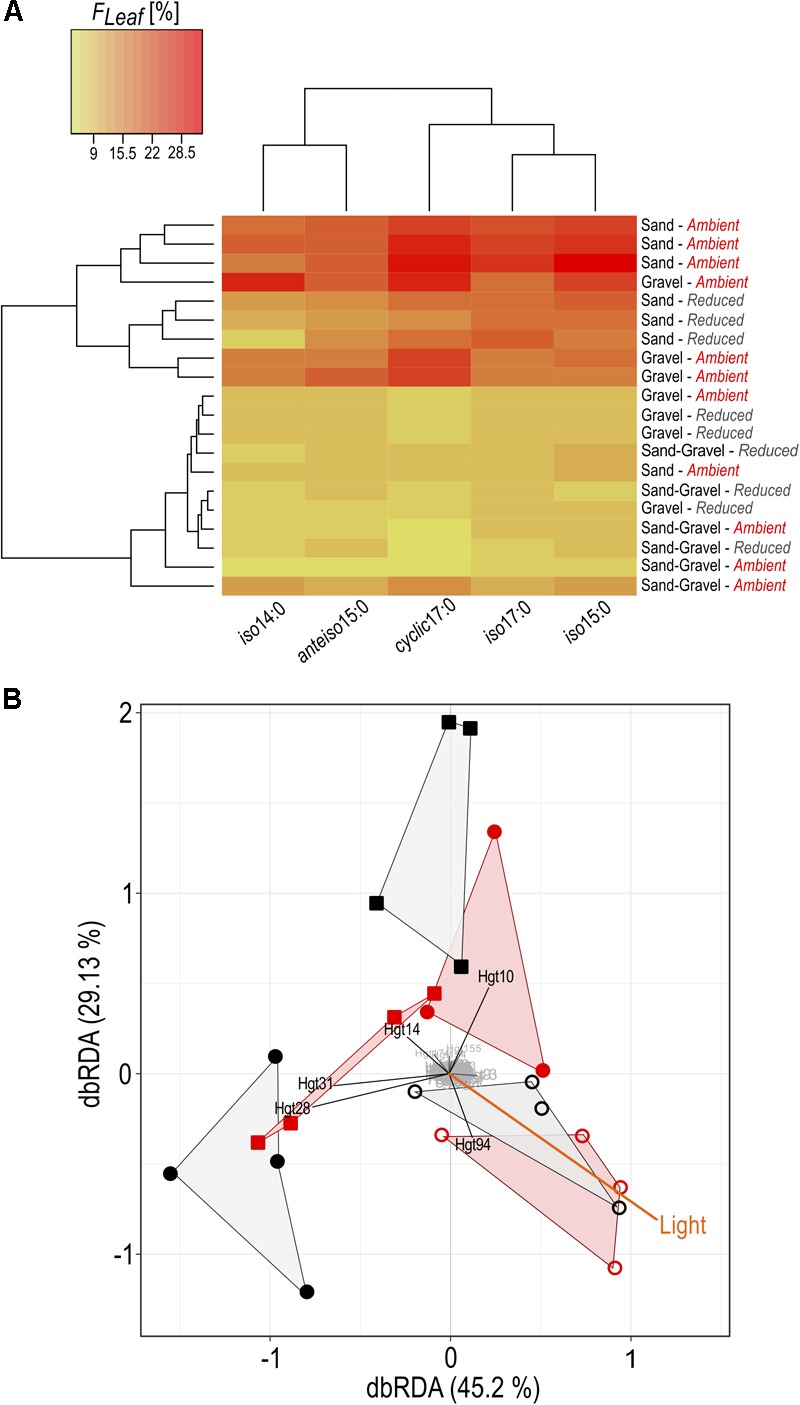
Alterations in bacterial community composition and C assimilation in relation to environmental conditions. **(A)** The proportion of leaf C in bacterial PLFA is given, expressed in %. All treatment replicates are presented on sediment level (sand, gravel, sand-gravel) sampled from streambeds incubated under reduced or ambient light. **(B)** Distance-based redundancy analysis of bacterial community fingerprints and environmental data. A hull connects replicates of sand (open circles), gravel (circles) and sand-gravel (squares) sediments incubated under reduced (black) or ambient light (red). T-RF’s with marginal influence on underlying differences between the samples are highlighted in gray.

In bacteria-specific PLFAs, *F_Leaf_* varied between the sediments. These differences were not equally pronounced under ambient than under reduced light conditions. Under ambient light conditions, the proportion of leaf C was lowest in PLFAs from sand-gravel sediments of the mixed streambeds and highest in sand and gravel sediments of the sorted streambeds. Under reduced light conditions, however, no significantly different *F_Leaf_* values were measured between gravel and sand-gravel sediments, with *F_Leaf_* values being significantly higher in PLFAs from sandy sediments. Consequently, light-induced variations in the proportion of leaf C on PLFA biomass were sediment-specific. In sand and gravel sediments, we found a generally higher proportion of leaf C under ambient than under reduced light conditions. Thereby, the *F_Leaf_* gap between both light treatments was most pronounced for bacterial PLFAs from gravel habitats. On the contrary, the proportion leaf C in bacterial PLFAs from sand-gravel sediments did not vary significantly between both light treatments. In addition, the extent to which our treatments induced variations in the proportion of leaf C in bacterial PLFAs differed between the different PLFA types (**Figure 5A**).

### Environmental Shifts in PLFA and T-RFLP Profiles

**FIGURE 6:**
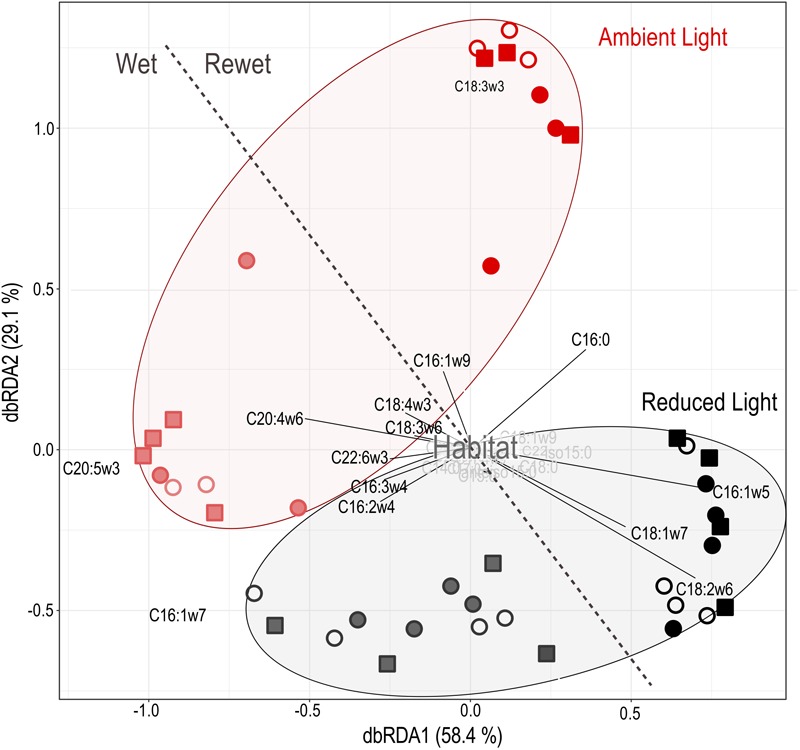
Distance-based redundancy analysis of PLFA profiles of sand (open circles), gravel (closed circles) and sand-gravel (squares) sediments and environmental data to assess compositional differences. Samples taken before (Wet = end of the colonization period) and after (Rewet = end of the rewet period) the drought period are separated by the dashed line. Environmental factors and PLFA types with marginal influence on underlying differences between the samples are highlighted in gray.

Distance based redundancy analysis (dbRDA) of PLFA profiles (**Figure 6**) indicated a significant shift in the composition of biofilm communities from pre- to post-drought conditions (*F* = 49.75, *P* < 0.001), which explained 40% of the variation in the data set. Additionally, PLFA profiles significantly varied according to light availability (*habitat: F* = 2.81, *P* = 0.001) but not among the sediment habitats (*habitat: F* = 1.19, *P* = 0.19). Combined results of PLFA and respiration analysis reveal that the rate of leaf C respiration was significantly correlated with light-induced shifts in PLFA profiles (*F* = 1.80, *P* = 0.03).

T-RFLP profiling of the bacterial 16S rRNA gene (**Figure 5B**) revealed highly diverse profiles of microbial communities, which varied considerably between replicate samples. In total, 289 T-RFs were identified after normalization. The profiles indicated significant variation in bacterial community structure in relation to treatment conditions (*F* = 1.58, *P* = 0.037). dbRDA revealed a greater influence of the sediment habitat (*habitat: F* = 1.29, *P* = 0.09) than light availability (*habitat × light: F* = 1.15, *P* = 0.24) on bacterial community structure, explaining 12.5 and 5.28% of variance in the data set, respectively. Distinct bacterial community composition in relation to light availability were only observed for gravel habitats (*habitat × light: F* = 1.37, *P* = 0.04). Microbial respiration of leaf C did not clearly correlate with the composition of the bacterial community (*Respiration-leaf C: F* = 1.29, *P* = 0.13, *F_Leaf_: F* = 0.81, *P* = 0.75). Nevertheless, the results indicate a significant relationship between leaf C respiration and light-induced shifts in the composition of the bacterial communities of gravel habitats (*F* = 2.16, *P* = 0.02), but not sandy habitats (*F* = 0.84, *P* = 0.73) and sand-gravel habitats (*F* = 0.69, *P* = 0.87).

## Discussion

In this study, we present evidence that streambed structure interacts with light as a driving factor for photo-heterotrophic interactions during leaf decomposition. Our results emphasize that the structure of the streambed defines the spatial availability of phototrophic metabolites in the streambed, as well as the composition of microbial streambed communities. Both are known to define the extent to which phototrophs and heterotrophs interact. Accordingly, our results emphasize that the structure of the streambed may influence the relevance of photo-heterotrophic interactions for leaf degradation. Furthermore, results confirm the fundamental influence of light availability and streambed structure on microbial functions in temporary streams.

### Interrelating Effects of Light and Streambed Structure on Microbial C Turnover

Phototrophic activities were strongly related to light availability as primary production was significantly higher under ambient than under reduced light conditions (see Zlatanović et al., 2017 for detailed discussion). Accordingly, we hypothesized that the decomposition of leaf C increases with increasing light availability because of a previously postulated stimulating effect of phototrophic activities on microbial leaf breakdown (Danger et al., 2013; Kuehn et al., 2014; Rier et al., 2014). Indeed, leaf C respiration rates were higher under ambient than under reduced light conditions, which agreed well with our expectations. On the contrary, stable isotope analyses of the respired C pool do not imply a generally promoted utilization of leaf C with increasing light availability, but instead highlight an interaction between our light and streambed treatments. While in sorted streams the proportion of leaf C in respired C was higher under ambient rather than reduced light conditions, in mixed streams the opposite was true. The stable isotope composition of the respired C pool and, consequently, the proportion of leaf C in the respired C pool, is the sum of heterotrophic and phototrophic activities (Fry and Sherr, 1989). At the ecosystem level, the phototrophic activities did not differ between the two streambed treatments (Zlatanović et al., 2017). Accordingly, the observed interactive effect of the streambed structure and the light availability on the composition of the respired C pool must underlie changes in the breakdown of leaf litter by heterotrophic activities. This assumption is further supported by the stable isotope analysis of bacterial PLFA biomarkers corresponding to the treatment-induced changes in the stable isotope ratios of the respired C pool. In gravel and sandy sediments (sorted streambeds), the higher proportion of leaf C in bacterial PLFA under ambient than under reduced light conditions indicates a higher utilization of leaf C by bacteria with increasing light availability. On the contrary, for bacterial PLFA from sand-gravel sediments (mixed streambeds), no significant differences between the light conditions were observed. Consequently, our results support that the streambed structure influences the proportional utilization of leaf C within the microbial food web, but also demonstrate that streambed structure had only a marginal influence on the magnitude of leaf C respiration. One possible explanation for these contradictory results could be the significant contribution of algae to leaf C respiration in our approach (Zlatanović et al., 2017). As previously mentioned, phototrophic and heterotrophic activities were not equally affected by our streambed treatments. Moreover, *F_Leaf_* values of the algae-specific fatty acid biomarkers varied differently according to light availability and streambed structure compared to bacteria-specific fatty acid biomarkers (**Figure 4**). Hence stable isotope analysis of fatty acids implies distinct effects of our treatments on the resource utilization of algae compared to bacteria. It is therefore possible that the high contribution of algae to C respiration superimposed the effect of our treatments on the magnitude of leaf C respiration by the heterotrophic community.

### Streambed Structure Determines the Spatial Availability of Algal Metabolites

While phototrophic activities and thus the production of energy-rich OM by phototrophs are limited to the benthic zone, heterotrophic processes occur throughout the entire streambed (Battin et al., 2016). Accordingly, heterotrophic activities are significantly influenced by hyporheic flow, as it determines the solute supply with regard to organic substrates, nutrients and redox partners (Battin et al., 2003; Perujo et al., 2017). We hypothesized that a greater hyporheic flow and resultant greater vertical solute transfer across the streambed would expand the stimulatory function of phototrophic metabolites on heterotrophic leaf decomposition to deeper sediment areas. Light-related changes in the assimilation of leaf C into bacterial PLFAs were most pronounced in gravel sediments compared to sandy and sand-gravel sediments (**Figures 4, 5A**). Gravel sediments are characterized by a much deeper photic zone and greater hydraulic conductivity than sand or sand-gravel sediments (Mendoza-Lera et al., 2017). Hence, consistent with our assumptions, the stronger hydraulic conductivity and the deeper benthic zone in gravel sediments could thus have favored the availability of algae metabolites into deeper sediment areas. Given the stimulating effect of algae metabolites on heterotrophic activities (Cole et al., 1982), this could have led to a greater proportion of the heterotrophic sediment community being mobilized in their activity, meaning that more leaf OM was actively degraded accompanied by a higher proportional use of leaf C within the microbial food web.

Our assumption that the streambed structure influences the effect of light on leaf C turnover by altering the spatial availability of algal metabolites is further supported by differences in leaf C utilization between sand and sand-gravel sediments. Indeed, both sediments provide similar habitat characteristics with respect to specific sediment surface area, a low depth of the photic zone, and low hydraulic conductivity, and thus share limited solute transport (Mendoza-Lera et al., 2017). Nevertheless, we observed stronger light-induced changes in the biomass proportion of leaf C in sandy than in sand-gravel habitats. Compared to findings by Mendoza-Lera et al. (2017), our VWE data indicated an increase in hydraulic conductivity in sandy sediments when sand and gravel were arranged in blocks (sorted streambed). Furthermore, in a related study, we demonstrate that the sorted arrangement of sand and gravel sediments provides a deeper vertical and lateral supply of benthic solutes from highly conductive gravel to low conductive sandy sediments (data unpublished). Accordingly, our findings provide evidence that a heterogeneous arrangement of high and low hydraulic sediments in the streambed enhances the effect of light on leaf degradation in low hydraulic sediments such as sand. In turn, our data underlines that recognized effects of light availability on leaf C turnover in microbial streambed biofilms are modified by the hyporheic flow in the streambed.

### Environmental Effects on Bacterial Community Composition

In addition to the spatial availability of algal metabolites, habitat conditions also influence the species composition of the microbial community (Zeglin, 2015). Indeed, T-RFLP profiling of bacterial DNA implies different species compositions among sand, gravel and sand gravel habitats (**Figure 5B**). Previous research emphasizes that bacterial species largely differ in their metabolic capability to degrade leaf C (Hutalle-Schmelzer et al., 2010; Carlson and Hansell, 2015). The differing leaf C utilization among the bacterial communities on sand, gravel, and sand-gravel sediments could therefore be due to a different species composition and hence metabolic capability. In addition, interactions between bacteria and algae are species-specific (Sarmento and Gasol, 2012; Horňák et al., 2017), which implies that only certain bacterial strains are affected in their functional role in leaf degradation by algal OM. Observed differences in *F_Leaf_* among bacterial PLFAs support this notion (**Figures 4, 5A**). Bacterial taxa produce the measured fatty acids in different proportions (Haack et al., 1994; Boschker et al., 1998). For example, *iso14:0* almost exclusively comprises in the membrane of the taxa *Actinobacteria* and *Firmicutes*, whereas *iso15:0* dominates in the membrane of *Bacterioidetes* species. Consequently, the observed interrelating effects of streambed structure and light availability on leaf C turnover could be linked to a different species occurrence within the communities in sand, gravel and sand-gravel habitats, which interact differently with algal OM. However, previous research also hints that induced changes in the function of a microbial community in carbon turnover underlie a change in the composition of the microbial community (Jones et al., 2009; Attermeyer et al., 2014; Blanchet et al., 2017). Accordingly, the light-induced changes in bacterial utilization of leaf C should have been accompanied by a change in community composition. This, however, cannot be fully proven by our measurements, as light had only an indirect influence on the composition of the T-RFLP profiles. Only for gravel sediments did we observe a significant shift in the composition of the community with varying light availability, which correlated well with the light-induced shift in leaf C utilization.

Consequently, PLFA and T-RFLP data suggested a species-related shift in bacterial function via photosynthetically produced OM and underline that communities are likely differentially stimulated by algal OM, depending on their species composition and/or additional habitat-related factors. Although environmental conditions may determine the composition of bacterial biofilm communities, light-induced shifts in microbial function did not clearly relate to shifts in community composition.

### Fundamental Importance of Light and Streambed Structure for the Functioning of Intermittent Streams

In view of the increasing proportion of intermittent stream ecosystems, it is important to understand how periodic drought alters environmental impacts on leaf decay. Recent findings emphasize that alternating wet–dry states disturb microbial activities with profound consequences for microbial function in the ecosystem C cycle (Febria et al., 2015). However, periodic desiccation of sediments has also been described to increase the bioavailability of leaf litter, thereby stimulating its microbial turnover (Abril et al., 2016; Weise et al., 2016). Indeed, drought disturbed microbial activity as discussed in detail by Zlatanović et al. (2017), accompanied by a lower respiration rate of leaf C (**Figure 2**). The decline in leaf C respiration accords well with previously reported adverse effects of drought on ecosystem function (Datry et al., 2014) associated with the death of the microbial cells from osmotic stress and consequently a reduction in microbial biomass (Birch, 1960; Rees et al., 2006). Hence, the observed reduction in biofilm activity from pre- to post-drought conditions likely resulted from an accompanying decrease in biofilm biomass. However, drought events have also been shown to affect microbial function through modification of community composition (Pohlon et al., 2013), which is in accordance with the differences in PLFA profiles between pre- and post-drought conditions observed in our study (**Figure 6**). Microbial species differ in their activity and thus recycle OM at different rates (Edwards and Meyer, 1986; Steward et al., 1996). Therefore, the combined effects of drought on biofilm biomass and community composition likely lead to a general reduction in biofilm activity and consequently, leaf C respiration. However, the effect of light and streamed structure on the proportion of leaf C in the DOC and the respired C pools (**Figure 3**) remained similar before and after the drought event. Hence, in contrast to previous results (Abril et al., 2016; Weise et al., 2016), the dryness of the streambed did not seem to further stimulate the decomposition of leaf C in our approach. Thus, despite the negative impact of the drought period on the magnitude of leaf C respiration, no interference of flow intermittency with the effect of our treatments on microbial utilization of leaf C was observed. In view of increasing drought periods in future, not only in streams but also in lakes and ponds, our results indicate that flow intermittency may not fully diminish the effects of environmental drivers of terrestrial-aquatic C cycling that were reported for perennial aquatic systems. It is necessary, however, to keep in mind that flow intermittency was mimicked by only one full dry–wet cycle in this study. Sediment drying reportedly most significantly affects phototrophic biofilm communities, depending on the intensity and frequency of drought events (Ledger et al., 2008; Timoner et al., 2012). It is therefore likely that in intermittent streams frequently exposed to drought, photo-heterotrophic interactions become less important for leaf C turnover with increasing intensity of drought stress.

## Conclusion

**FIGURE 7:**
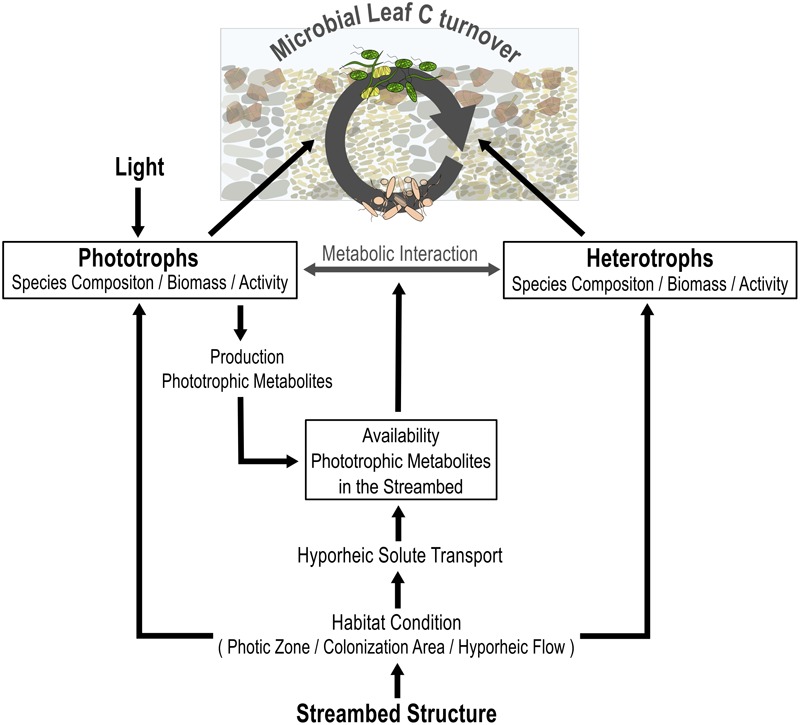
Conceptual overview of how light availability and streambed structure interactively modify the contribution of microbial phototrophs and heterotrophs to leaf C turnover, including their interaction. The availability of light controls the contribution of biofilm phototrophs to C turnover and, in turn, the production of algal metabolites in the streambed. Streambed structure defines the contribution of both, the phototrophic and the heterotrophic community, to leaf C turnover by defining habitat conditions in terms of hyporheic flow, light penetration depth, and available colonization area. The hyporheic flow, in turn, defines the spatial availability of algal metabolites in the streambed.

In summary, the results from this study highlight that the availability of light and the structure of the streambed interactively modulate the cycling of leaf C within the microbial food web (**Figure 7**). Light availability determined the occurrence and thus functional importance of algae to leaf C cycling, including the stimulatory effect of algal metabolites on the activity of heterotrophic bacteria. Our results further suggest that the streambed structure determines the spatial availability of algal exudates within the streambed, as well as the species composition of bacterial communities. That in turn, defines the stimulatory function of phototrophic activities for the use of leaf C by heterotrophic bacteria. Accordingly, our results emphasize that environmental factors interactively influence the relevance of photo-heterotrophic interactions for leaf degradation.

In addition, our results highlight a prominent influence of light and streambed structure on microbial leaf C turnover, even under dynamic hydrological conditions. This, in turn, underlines the fundamental role of both environmental factors in the functioning of stream ecosystems.

Collectively, the results from this study provide valuable insights for future studies on the mechanisms by which environmental factors control terrestrial C turnover in stream ecosystems, particularly regarding the ecological functions of photo- and heterotrophic biofilm communities. Given the multitude of environmental factors potentially affecting biofilm C metabolism, in addition to invertebrates and protist grazers, we emphasize the need for additional *in situ* surveys to better estimate the relevance of photo-heterotrophic coupling for terrestrial C turnover in natural ecosystems.

## Author Contributions

The study was designed and conducted by SZ, KP, MM, and JF. Stable isotope analysis of PLFA, respired C, DOC, and particulate OM, including data interpretation, was performed by RvG, GG, and JF. T-RFLP of bacterial DNA, including data interpretation, was performed by AU and JF. The combined data set was interpreted and discussed by KP, H-PG, and JF. The manuscript was written by JF. KP, MM, H-PG, SZ, AU, and RvG contributed to the writing and correction of the manuscript. All authors read and approved the final manuscript.

## Conflict of Interest Statement

The authors declare that the research was conducted in the absence of any commercial or financial relationships that could be construed as a potential conflict of interest.
